# 血浆miR-34b-3p和miR-302a-5p在非小细胞肺癌诊断中的临床应用

**DOI:** 10.3779/j.issn.1009-3419.2019.04.03

**Published:** 2019-04-20

**Authors:** 治鹏 宋, 宗德 张, 洋 刘

**Affiliations:** 1 101149 北京，首都医科大学附属北京胸科医院流行病研究室 Department of Epidemiology; Beijing Chest Hospital, Capital Medical University, Beijing 101149, China; 2 101149 北京，首都医科大学附属北京胸科医院分子生物学实验室 Laboratory of Molecular Biology, Beijing Chest Hospital, Capital Medical University, Beijing 101149, China

**Keywords:** 肺肿瘤, 诊断, 微小核糖核酸, 肿瘤标志物, Lung neoplasms, Diagnosis, microRNA, Tumor markers

## Abstract

**背景与目的:**

微小核糖核酸（microRNA, miRNA）是一类长约22个核苷酸的单链非编码RNA，其表达异常与疾病密切相关。本研究旨在探讨miR-34b-3p和miR-302a-5p在非小细胞肺癌（non-small cell lung cancer, NSCLC）患者血浆中的表达水平及其在NSCLC诊断的临床应用价值研究。

**方法:**

采用荧光定量PCR（real-time polymerase chain reaction, RT-PCR）对86例NSCLC患者、64例肺结核（pulmonary tuberculosis, PTB）患者、39例健康体检者血浆中的miR-34b-3p和miR-302a-5p含量进行测定，同时对比并结合癌胚抗原（carcino-embryonic antigen, CEA）、神经元烯醇化酶（neuron-specific enolase, NSE）和血清骨胶素（cytokeratin 19 fragments 21-1, CYFRA21-1），分析其诊断NSCLC的价值。

**结果:**

NSCLC组血浆中miR-34b-3p及miR-302a-5p表达水平明显高于PTB组和健康对照组（*P* < 0.05）。血浆miR-34b-3p的表达水平与肿瘤直径相关（*P* < 0.01）。单项检测NSCLC时，miR-302a-5p的灵敏度最高（82.6%），CEA的特异度最高（81.6%）。双项检测NSCLC时，miR-34b-3p+miR-302a-5p的灵敏度最高（80.2%），miR-34b-3p+CEA的特异度最高（89.3%）。多项检测NSCLC时，miR-302a-5p+NSE+CYFRA21-1的灵敏度最高（81.4%），miR-34b-3p+CEA+ NSE的特异度最高（90.3%）。当联合miR-34b-3p、miR-302a-5p和CEA进行检测时，其ROC曲线下面积AUC（area under the curve, AUC）最大，为0.832。*Logistic*回归模型表明：在NSCLC与对照组（PTB组+健康对照组）中，miR-34b-3p可能是NSCLC发生的独立危险因素。

**结论:**

血浆miR-34b-3p、miR-302a-5p有可能成为NSCLC辅助诊断的血浆学标志物。

## Abstract (en)

**Background and objective:**

MicroRNA is a kind of single-stranded non-coding RNA whose length is about 22 nucleotides and its abnormal expression is related to disease closely. This study is aiming to explore the relative expression of miR-34b-3p and miR-302a-5p in the plasma of non-small cell lung cancer (NSCLC) patients and its clinical value.

**Methods:**

The levels of miR-34b-3p and miR-302a-5p in plasma were detected by real-time polymerase chain reaction (RT-PCR) in 86 patients with NSCLC, 64 patients with pulmonary tuberculosis (PTB) and 39 healthy subjects. Analyze their value in diagnosing NSCLC by contrasting and combining carcino-embryonic antigen (CEA), neuron-specific enolase (NSE), and cytokeratin 19 fragments 21-1 (CYFRA21-1).

**Results:**

The levels of plasma miR-34b-3p and miR-302a-5p in NSCLC group were significantly higher than those in the PTB group and the healthy group (*P* < 0.05). In patients with NSCLC, the levels of plasma miR-34b-3p was correlated with the diameter of tumor (*P* < 0.01). When using one plasma marker to diagnose NSCLC, miR-302a-5p had the highest sensitivity (82.6%) and CEA had the highest specificity (81.6%). While combined two plasma markers, miR-34b-3p+miR-302a-5p had the highest sensitivity (80.2%) and miR-34b-3p+CEA had the highest specificity (81.4%). As detected multiple markers, miR-302a-5p+NSE+CYFRA21-1 had the highest sensitivity (81.4%) and miR-34b-3p+CEA+NSE had the highest specificity (90.3%). The combination of miR-34b-3p, miR-302a-5p and CEA obtained the highest area under the curve (AUC), which was 0.832. *Logistic* regression model indicated that miR-34b-3p was independent risk factor for NSCLC compared to control groups.

**Conclusion:**

Plasma miR-34b-3p and miR-302a-5p could be used as biological markers for the diagnosis of NSCLC.

肺癌是最常见的恶性肿瘤之一，近年来其发病率和死亡率在全球范围内明显增加^[1, 2]^，且5年内存活率 < 15%，尽管有一些化疗及手术等手段，存活率仍 < 60%^[3]^。目前，肺癌的临床诊断主要依靠影像学检查、临床指征分析和组织活检^[4]^等，但以上方法均存在一定的局限性，尚无敏感性和特异性较高的检测手段。血液肿瘤标志物可以用于疑似肺癌患者的初步诊断判断，但现有的肺癌血液肿瘤标志物[癌胚抗原（carcino-embryonic antigen, CEA）、糖类抗原199（carbohydrate atigen 19-9, CA199）、糖类抗原125（carbohydrate atigen 125, CA125）、神经元烯醇化酶（neuron-specific enolase, NSE）、血清骨胶素（cytokeratin 19 fragments 21-1, CYFRA21-1）、SCC等]虽然被广泛地应用，但其在非肿瘤疾病中也会存在异常表达，因此，CEA等肿瘤标志物用于肺癌诊断的敏感性较差。

微小核糖核酸（microRNA, miRNA）是一类长约22个核苷酸的单链非编码RNA，其表达异常与疾病密切相关^[5]^，具有成为多疾病尤其是肿瘤诊断标志物的潜能^[6, 7]^。本项研究在前期通过关键词[lung cancer and（microRNA or miR）and（serum or plasma）]检索，整合基因表达数据库（gene expression omnibus, GEO）中获得的不同的数据（GSE68951、GSE16512、GSE46729、GSE67804、GSE70080、GSE76462），挑选在多个数据中出现并且差异表达较高的指标。其中，miR-34b-3p在多种恶性肿瘤（前列腺癌、乳腺癌、子宫颈癌、肺癌）中表达异常，有望成为恶性肿瘤的生物学标志物或治疗靶点^[8-11]^；miR-302a异常表达与与卵巢癌^[12]^、急性髓细胞白血病（acute myeloid leukemia, AML）^[13]^、胃癌^[14]^的发生均密切相关，但在肺癌患者中的表达情况至今未见报道。以此为依据，最终确定所要研究的miRNA（miR-34b-3p和miR-302a-5p）。

本项研究通过检测血浆中miR-34b-3p和miR-302a-5p在非小细胞肺癌（non-small cell lung cancer, NSCLC）患者中的表达水平，分析其表达情况与临床病理特征之间的关系及其在NSCLC诊断的潜在价值，以期为NSCLC的诊断提供新思路和新依据。

## 1 资料与方法

### 1.1 一般资料

选取2018年6月-2018年10月首都医科大学附属北京胸科医院收治并确诊的NSCLC患者86例作为肺癌组，其中男性54例，女性32例，平均年龄（61.35±8.52）岁。纳入标准：①经组织病理学或细胞学诊断明确；②临床资料完整，包括人口学特征，如年龄、性别、吸烟状况、职业暴露、病理诊断、肿瘤-淋巴结-转移（tumor-node-metastasis, TNM）；③在本医院进行首程治疗；④未合并其他肿瘤或器官功能障碍性疾病。排除标准：①年龄 > 80岁；②肺内合并活动性炎症。选取同期收治并确诊为PTB的患者64例作为良性对照组，其中男性44例，女性20例，平均年龄（59.02±11.19）岁，诊断标准参照中华人民共和国国家卫生和计划生育委员会肺结核诊断标准（WS288-2017）。同期进行健康体检的39例作为健康对照组，其中男性24例，女性15例，平均年龄（57.32±9.18）岁。各组的性别、年龄等一般资料比较，差异均无统计学意义（*P* > 0.05），具有可比性。所有受试者均签署知情同意书。

### 1.2 方法

#### 1.2.1 标本采集

清晨空腹采集静脉血5 mL置于EDTA抗凝管中，所有血液自收集至处理时间不超过2 h。血液样本在4 ℃下、4, 000 rpm离心10 min；取上层血浆置于新的1.5 mL RNase-Free的离心管中，处理后的血浆于-80 ℃保存。

#### 1.2.2 miRNA的提取

冻存的样本室温融解后，4 ℃，16, 000 g离心10 min，取上层血浆200 μL。miRNA的提取严格按照商品化试剂盒（miRcute血清/血浆miRNA提取分离试剂盒，TIANGEN）的操作步骤进行。提取的microRNA加入25 μL的RNase-Free水溶解，使用NanoDrop2000微量分光光度计测定其浓度与纯度，而后直接进行miRNA的反转录。

#### 1.2.3 miRNA检测

用反转录试剂盒（miRcute增强型miRNA cDNA第一链合成试剂盒，TIANGEN）进行反转，其反应体系包括miRNA 8 μL，反转录缓冲液10 μL，反转录酶混合液2 μL；反转条件为42 ℃ 60 min、95 ℃ 3 min，4 ℃ 5 min。取反转录产物5 μL加45 μL RNase-Free水稀释。采用商品化试剂盒[miRcute增强型miRNA荧光定量检测试剂盒（SYBR Green），TIANGEN]进行荧光定量PCR（real-time polymerase chain reaction, RT-PCR）检测，其反应体系包括预混液10 μL，正向引物0.4 μL，反向引物0.4 μL，取稀释后的反转录产物2 μL，ROX染料2 μL，加5.2 μL RNase-Free水补足至20 μL。设置空白对照，所有反应采用3个复孔，使用QuantStudio 7荧光定量PCR仪（美国，Thermo Fisher Scientific）按照95 ℃ 15 min、5个预循环（94 ℃ 20 s、64 ℃ 30 s、72 ℃ 34 s）、40个循环（94 ℃ 20 s、60 ℃ 34 s）进行扩增。采用相对定量法对血浆中各miRNA含量进行分析。以U6为内参，2^-△Ct^作为其相对含量，其中△Ct=Ct_miRNA_-Ct_U6_。

### 1.3 统计学方法

数据采用IBM SPSS 24.0软件进行分析和处理。非正态分布的计量资料以中位数（四分位间距）[M（Q1, Q3）]表示，多组间比较采用*Kruskal-Wallis*检验，两组间血浆中miRNA表达水平的比较采用*Mann-Whitney U*检验。通过受试者工作特征曲线（receiver operator characteristic curve, ROC curve）分析miR-34b-3p和miR-302a-5p以及肿瘤标志物的诊断价值，获得临界值（cut-off point）以及灵敏度和特异度。使用*Logistic*回归分析miR-34b-3p和miR-302a-5p与NSCLC发生的相关性。以*P* < 0.05为差异有统计学意义。

## 2 结果

### 2.1 多组血浆miR-34b-3p和miR-302a-5p表达水平比较

结果显示miR-34b-3p、miR-302a-5p在NSCLC组、PTB组以及健康对照组中的表达水平存在差异，并且具有统计学意义（*P* < 0.01），见表 1。

**1:** 多组间血浆miRNAs表达水平比较[M（Q1, Q3）] Comparison of plasma miRNAs expression levels among multiple groups [M(Q1, Q3)]

miRNAs	NSCLC (*n*=86)	PTB (*n*=64)	Healthy control (*n*=39)	*P*
miR-34b-3p	0.252 (0.126, 0.447）	0.114 (0.037, 0.262)	0.098 (0.029, 0.242)	< 0.000, 1
miR-302a-5p	0.025 (0.008, 0.047)	0.012 (0.003, 0.033)	0.007 (0.003, 0.015)	0.000, 2

对miR-34b-3p、miR-302a-5p在NSCLC组和PTB组、NSCLC组和健康对照组中的表达情况，进行两组间的比较分析。结果显示miR-34b-3p和miR-302a-5p的表达在NSCLC组与PTB组以及NSCLC组与健康对照组中存在差异（*P* < 0.05），见表 2。

**2:** 两组间血浆miRNAs表达水平比较[M（Q1, Q3）] Comparison of plasma miRNAs expression levels between the two groups [M(Q1, Q3)]

miRNAs	NSCLC(*n*=86)	PTB(*n*=64)	*P*	Healthy control(*n*=39)	*P*
miR-34b-3p	0.252 (0.126-0.447)	0.114 (0.037, 0.262)	0.000, 1	0.098 (0.029, 0.242)	< 0.000, 1
miR-302a-5p	0.025 (0.008-0.047)	0.012 (0.003, 0.033)	0.015, 1	0.007 (0.003, 0.015)	< 0.000, 1

### 2.2 NSCLC患者血浆miR-34b-3p和miR-302a-5p表达与临床病理特征的关系

结果显示血浆miR-34b-3p表达水平与肿瘤直径相关，差异有统计学意义（*P* < 0.05），与年龄、性别、吸烟状况、职业暴露、病理分期、病理类型、淋巴结转移无关；而miR-302a-5p表达水平与年龄、性别、吸烟状况、职业暴露、病理分期、病理类型、淋巴结转移、肿瘤直径无关，差异无统计学意义（*P* > 0.05），见表 3。

**3:** 非小细胞肺癌患者血浆miRNAs表达与临床病理特征的关系 The relationship between plasma miRNAs expression and clinicopathological features in patients with non-small cell lung cancer

Clinical parameter		*n*	miR-34b-3p			miR-302a-5p	
			[M (Q1, Q3)]	*P*		[M(Q1, Q3)]	*P*
Age (year)	≤60	38	0.261 (0.114, 0.498)	0.791, 9		0.034 (0.008, 0.056)	0.413, 2
	> 60	48	0.240 (0.137, 0.391)			0.020 (0.010, 0.044)	
Gender	Male	54	0.316 (0.134, 0.516)	0.191, 7		0.032 (0.010, 0.052)	0.154, 9
	Female	32	0.201 (0.112, 0.326)			0.018 (0.008, 0.040)	
Smoking state	Yes	42	0.286 (0.144, 0.554)	0.113, 2		0.030 (0.010, 0.055)	0.226, 1
	No	44	0.210 (0.084, 0.388)			0.019 (0.008, 0.041)	
Occupational exposure	Yes	3	0.508 (0.161, 0.694)	0.293, 7		0.035 (0.003, 0.066)	0.876, 8
	No	83	0.249 (0.115, 0.435)			0.024 (0.009, 0.047)	
Pathological stages	Ⅰ-Ⅱ	52	0.195 (0.111, 0.414)	0.085, 8		0.027 (0.007, 0.045)	0.613, 4
	Ⅲ-Ⅳ	34	0.327 (0.158, 0.516)			0.023 (0.011, 0.051)	
Pathological types	Adenocarcinoma	56	0.260 (0.113, 0.461)	0.623, 3		0.018 (0.009, 0.047)	0.468, 0
	Squamous cell carcinoma	30	0.231 (0.141, 0.429)			0.033 (0.007, 0.052)	
Lymph node metastasis	Yes	36	0.327 (0.147, 0.549)	0.145, 6		0.030 (0.012, 0.051)	0.136, 1
	No	50	0.208 (0.113, 0.390)			0.019 (0.007, 0.041)	
Diameter of tumor (cm)	≤5	75	0.208 (0.114, 0.389)	0.009, 8		0.021 (0.008, 0.046)	0.114, 3
	> 5	15	0.473 (0.255, 0.731)			0.042 (0.017, 0.066)	

### 2.3 血浆miR-34b-3p和miR-302a-5p单独及与肿瘤标志物联合检测对NSCLC的诊断价值

ROC曲线用来评估血浆miRNA对NSCLC的诊断价值。结果显示，单项检测NSCLC时，miR-302a-5p的灵敏度最高（82.6%），CEA的特异度最高（81.6%）；双项检测NSCLC时，miR-34b-3p+miR-302a-5p的灵敏度最高（80.2%），miR-34b-3p+CEA的特异度最高（89.3%）；多项检测NSCLC时，miR-302a-5p+NSE+CYFRA21-1的灵敏度最高（81.4%），miR-34b-3p+CEA+NSE的特异度最高（90.3%）。当联合miR-34b-3p、miR-302a-5p和CEA这三项进行检测时，其ROC曲线下面积（area under the curve, AUC）最大，为0.832。见表 4和图 1。

**1:**
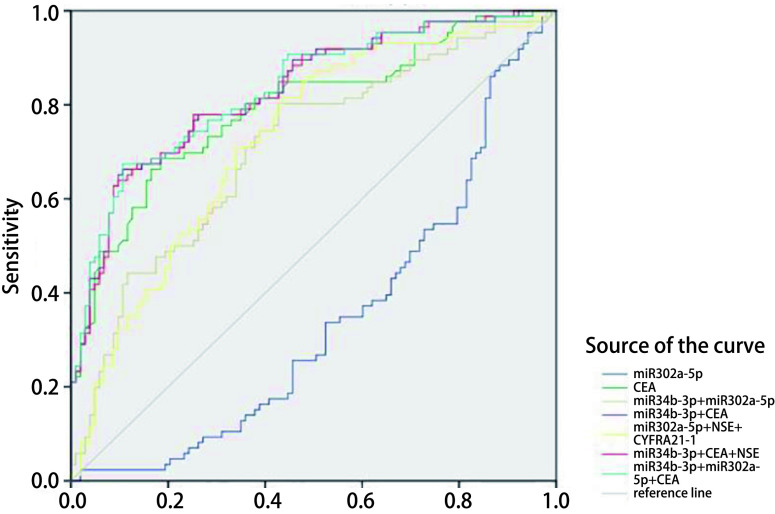
miR-34b-3p、miR-302a-5p以及肿瘤标志物的ROC曲线 ROC curve of miR-34b-3p, miR-302a-5p and tumor markers. ROC: receiver operating characteristic.

**4:** 血浆miR-34b-3p、miR-302a-5p、肿瘤标志物对非小细胞肺癌的诊断价值 Diagnostic value of plasma miR-34b-3p, miR-302a-5p and tumor markers for non-small cell lung cancer

Parameter	AUC(95%CI)	*P*	Sensitivity(%)	Specificity(%)	Cut-off point
One marker					
miR-34b-3p	0.703 (0.628-0.778)	< 0.001	81.4%	55.3%	0.367
miR-302a-5p	0.660 (0.582-0.737)	< 0.001	82.6%	44.7%	0.273
CEA	0.793 (0.727-0.858)	< 0.001	68.6%	81.6%	0.502
NSE	0.608 (0.527-0.689)	0.011	62.8%	61.2%	0.240
CYFRA21-1	0.759 (0.690-0.828)	< 0.001	72.1%	73.8%	0.459
Two markers					
miR-34b-3p+CEA	0.829 (0.770-0.888)	< 0.001	66.3%	89.3%	0.556
miR-34b-3p+NSE	0.696 (0.621-0.771)	< 0.001	47.7%	85.4%	0.331
miR-34b-3p+CYFRA21-1	0.749 (0.679-0.818)	< 0.001	58.1%	83.5%	0.416
miR-302a-5p+CEA	0.792 (0.726-0.857)	< 0.001	68.6%	81.6%	0.502
miR-302a-5p+NSE	0.615 (0.534-0.696)	0.007	64.0%	62.1%	0.261
miR-302a-5p+CYFRA21-1	0.749 (0.679-0.820)	< 0.001	68.6%	75.7%	0.443
miR-34b-3p+miR-302a-5p	0.709 (0.634-0.783)	< 0.001	80.2%	57.3%	0.375
Multiple markers					
miR-34b-3p+CEA+NSE	0.828 (0.769-0.887)	< 0.001	64.0%	90.3%	0.543
miR-34b-3p+CEA+CYFRA21-1	0.828 (0.768-0.887)	< 0.001	66.3%	89.3%	0.556
miR-34b-3p+NSE+CYFRA21-1	0.734 (0.663-0.805)	< 0.001	58.1%	80.6%	0.387
miR-34b-3p+CEA+NSE+CYFRA21-1	0.826 (0.767-0.885)	< 0.001	66.3%	87.4%	0.537
miR-302a-5p+CEA+NSE	0.793 (0.727-0.859)	< 0.001	68.6%	81.6%	0.502
miR-302a-5p+CEA+CYFRA21-1	0.791 (0.725-0.857)	< 0.001	69.8%	80.6%	0.504
miR-302a-5p+NSE+CYFRA21-1	0.725 (0.653-0.797)	< 0.001	81.4%	56.3%	0.377
miR-302a-5p+CEA+NSE+CYFRA21-1	0.789 (0.722-0.855)	< 0.001	69.8%	80.6%	0.504
miR-34b-3p+miR-302a-5p+CEA	0.832 (0.774-0.890)	< 0.001	67.4%	89.3%	0.567
miR-34b-3p+miR-302a-5p+NSE	0.703 (0.629-0.778)	< 0.001	46.5%	87.4%	0.339
miR-34b-3p+miR-302a-5p+CYFRA21-1	0.742 (0.672-0.813)	< 0.001	55.8%	85.4%	0.412
miR-34b-3p+miR-302a-5p+CEA+NSE	0.832 (0.774-0.890)	< 0.001	67.4%	89.3%	0.567
miR-34b-3p+miR-302a-5p+CEA+CYFRA21-1	0.830 (0.771-0.888)	< 0.001	67.4%	89.3%	0.567
miR-34b-3p+miR-302a-5p+NSE+CYFRA21-1	0.733 (0.661-0.804)	< 0.001	58.1%	82.5%	0.406
miR-34b-3p+miR-302a-5p+CEA+NSE+CYFRA21-1	0.830 (0.772-0.889)	< 0.001	67.4%	89.3%	0.567

### 2.4 *Logistic*回归将miR-34b-3p、miR-302a-5p进行单因素*Logistic*回归分析

结果显示在NSCLC组与对照组（PTB组+健康对照组）中，miR-34b-3p的优势比（odds ratio, OR）为9.214（*P* < 0.05），说明其具有一定的预测性，可能为NSCLC发病的独立危险因素。见表 5。

**5:** 单因素*Logistic*回归 Univariate *Logistic* regression

miRNAs	NSCLC group *vs* Control group		
	OR	95%CI	*P*
miR-34b-3p	9.214	2.442-34.768	0.001
miR-302a-5p	0.963	0.83-1.118	0.622

## 3 讨论

在临床上，新的生物标志物是肺癌患者的诊断、指导治疗和监测肺部病变的关键^[15, 16]^。基因组学和蛋白质组学技术的飞速进展加快了许多潜在的临床应用标志物的发现。血液学检查对患者创伤小且操作简便，因此在血液中找到合适的分子标志物，阐明其与肺癌发生的关系，显得尤为重要^[17]^。miRNA是一种非编码的小分子单链RNA，是调控基因在转录后及翻译水平表达的主要元件^[18]^，报道表明miRNA与肿瘤、炎症等许多疾病的发生、发展等密切相关，且miRNA取材方便，在血浆中稳定存在，可连续监测，有望成为一种理想的潜在血浆肿瘤标志物^[19, 20]^。

本研究发现miR-34b-3p和miR-302a-5p，与对照组（PTB组+健康对照组）相比，两者在NSCLC血浆中均高表达，表示其在肺癌的发生发展过程中可能起正向调控作用。其中miR-34b-3p的表达与既往研究结果相一致^[21]^，并且还发现其表达水平与肿瘤直径相关，在肿瘤直径 > 5 cm的患者血浆中的表达水平明显高于肿瘤直径≤5 cm的患者。分析其原因可能是肿瘤负荷越大，肿瘤组织释放具有原癌基因作用的miRNA进入血液的机会越大，使得机体原有的具有抑癌作用的miRNA表达水平受到抑制，进而导致机体miRNA表达水平异常升高。由此可见，miR-34b-3p在一定程度上参与NSCLC的发生发展。然而miR-34b-3p的表达在年龄、性别、吸烟状况、职业暴露、病理分期、病理类型、淋巴结转移分类中却未发现差异。分析其原因可能是因为本次研究的样本量相对较小，仍需进一步扩大，才能验证miRNA含量在不同分组中的表达差异。我们还将miR-34b-3p进行了单因素*Logistic*回归分析，结果显示在NSCLC组与对照组（PTB组+健康对照组）中，miR-34b-3p的OR值为9.214^[22]^。由于对入组患者的相关随访还在进行中，未进行*Cox*回归等相关分析。因此，miR-34b-3p可能是NSCLC发生的独立危险因素，但仍需进一步验证。

关于miR-302a-5p与肺癌之间的相关研究至今未见报道，参考其在卵巢癌、急性髓细胞白血病（acute myeloid leukemia, AML）、胃癌中的表达情况，发现miR-302a-5p在卵巢癌和AML细胞中高表达^[12, 13]^，在胃癌组织中低表达^[14]^。本研究中miR-302a-5p的表达情况与其在卵巢癌和AML中表达相一致。在分析其表达水平与临床病理特征的关系时，却未发现两者之间的关联。造成这种结果可能的原因，一是受限于本次研究的样本量；二是受限于纳入研究的病理特征的分类，其表达水平有可能在其他病理特征分类上存在统计学差异。

我们通过计算ROC曲线下面积AUC，来评估miR-34b-3p、miR-302a-5p及联合肿瘤标志物对NSCLC的诊断价值。有研究组合6种miRNA（miR-429、miR-205、miR-200b、miR-203、miR-125b、miR-34b）对早期NSCLC进行诊断，获得的AUC为0.88^[21]^。由此可以肯定miR-34b在NSCLC早期诊断中的价值。本研究发现，检测86例NSCLC（Ⅰ期-Ⅱ期52例，Ⅲ期-Ⅳ期34例）血浆中miR-34b-3p、miR-302a-5p的表达，获得的曲线下面积分别为0.703和0.660，并且可以获得≥81.4%的诊断灵敏度，但特异度并不理想（最大值为55.3%），这为miR-34b用于NSCLC诊断方面提供了更为全面的证据，并且探索了miR-302a-5p用于NSCLC诊断的价值。应用肿瘤标志物CEA、NSE、CYFR21-1对入组病例进行诊断可以获得较好的诊断特异度（≥61.2%），但是敏感度较差（最大值为72.1%）。由此，本研究尝试将血浆中miRNA检测与肿瘤标志物检测结果结合，以此来提高诊断效能^[23]^。发现多项检测NSCLC时，miR-302a-5p+NSE+CYFRA21-1的灵敏度最高（81.4%），miR-34b-3p+CEA+NSE的特异度最高（90.3%）。当联合miR-34b-3p、miR-302a-5p和CEA进行检测时，其AUC最大，为0.832。由此可见，血浆miR-34b-3p、miR-302a-5p与肿瘤标志物联合，有利于提高其对NSCLC的诊断灵敏度、特异度以及诊断效能，具有潜在的应用价值。

综上所述，NSCLC患者血浆中miR-34b-3p和miR-302a-5p的表达水平高于对照人群，并且通过组合miRNA与肿瘤标志物，可以获得满意的诊断效能，有望成为NSCLC辅助诊断的血浆学标志物，为液体活检的发展提供了新的思路。

## References

[1] Torre LA, Bray F, Siegel RL (2015). Global cancer statistics, 2012. CA Cancer J Clin.

[2] Bray F, Ferlay J, Soerjomataram I (2018). Global cancer statistics 2018: GLOBOCAN estimates of incidence and mortality worldwide for 36 cancers in 185 countries. CA Cancer J Clin.

[3] Herbst RS, Heymach JV, Lippman SM (2008). Lung cancer. N Engl J Med.

[4] Diaz LA, J r., Bardelli A. (2014). Liquid biopsies: genotyping circulating tumor DNA. J Clin Oncol.

[5] Lv JJ, Xu L, Xu YT (2014). Expression of MiRNA-221 in non-small cell lung cancer tissues and correlation with prognosis. Zhongguo Fei Ai Za Zhi.

[6] Montani F, Marzi MJ, Dezi F (2015). miR-test: a blood test for lung cancer early detection. J Natl Cancer Inst.

[7] Song ZP, Liu Y (2018). Progress of liquid biopsy in early diagnosis of lung cancer. Zhongguo Fei Ai Za Zhi.

[8] Fang LL, Sun BF, Huang LR (2017). Potent inhibition of miR-34b on migration and invasion in metastatic prostate cancer cells by regulating the TGF-beta pathway. Int J Mol Sci.

[9] Kim YH, Lee WK, Lee EB (2017). Combined effect of metastasis-related microRNA, miR-34 and miR-124 family, methylation on prognosis of non-small-cell lung cancer. Clin Lung Cancer.

[10] Sanaei S, Hashemi M, Rezaei M (2016). Evaluation of the pri-miR-34b/c rs4938723 polymorphism and its association with breast cancer risk. Biomed Rep.

[11] Yuan F, Sun R, Chen P (2016). Combined analysis of pri-miR-34b/c rs4938723 and TP53 Arg72Pro with cervical cancer risk. Tumour Biol.

[12] Guo T, Yu W, Lv S (2015). MiR-302a inhibits the tumorigenicity of ovarian cancer cells by suppression of SDC1. Int J Clin Exp Pathol.

[13] Liu X, Heng C, Li Y (2017). MiR-302a sensitizes leukemia cells to etoposide by targeting Rad52. Oncotarget.

[14] Ma G, Li Q, Dai W (2017). Prognostic implications of miR-302a/b/c/d in human gastric cancer. Pathol Oncol Res.

[15] Vargas AJ, Harris CC (2016). Biomarker development in the precision medicine era: lung cancer as a case study. Nat Rev Cancer.

[16] Villalobos P, Wistuba II (2017). Lung cancer biomarkers. Hematol Oncol Clin North Am.

[17] Matikas A, Syrigos KN, Agelaki S (2016). Circulating biomarkers in non-small-cell lung cancer: current status and future challenges. Clin Lung Cancer.

[18] Hou J, Meng F, Chan LW (2016). Circulating plasma microRNAs as diagnostic markers for NSCLC. Front Genet.

[19] He Y, Lin J, Kong D (2015). Current state of circulating microRNAs as cancer biomarkers. Clin Chem.

[20] Mitchell PS, Parkin RK, Kroh EM (2008). Circulating microRNAs as stable blood-based markers for cancer detection. Proc Natl Acad Sci U S A.

[21] Halvorsen AR, Bjaanaes M, LeBlanc M (2016). A unique set of 6 circulating microRNAs for early detection of non-small cell lung cancer. Oncotarget.

[22] Sha J, Fan LH (2018). Diagnostic value of miRNA-146b-3p in early non-small cell lung cancer. Tongji Da Xue Xue Bao (Yixueban).

[23] He J, Xiao B, Hang JF (2018). Clinical application of serum miR-122-5p and miR486-5p in the diagnosis of hepatocellular carcinoma. Zhonghua Jian Yan Yi Xue Za Zhi.

